# Socioeconomic Determinants of Multimorbidity: A Population-Based Household Survey of Hong Kong Chinese

**DOI:** 10.1371/journal.pone.0140040

**Published:** 2015-10-09

**Authors:** Roger Y. Chung, Stewart Mercer, Francisco T. T. Lai, Benjamin H. K. Yip, Martin C. S. Wong, Samuel Y. S. Wong

**Affiliations:** 1 School of Public Health and Primary Care, Faculty of Medicine, The Chinese University of Hong Kong, Hong Kong, China; 2 General Practice and Primary Care, Institute of Health and Wellbeing, University of Glasgow, Glasgow, Scotland, United Kingdom; University of Brescia, ITALY

## Abstract

**Introduction:**

Multimorbidity has been well researched in terms of consequences and healthcare implications. Nevertheless, its risk factors and determinants, especially in the Asian context, remain understudied. We tested the hypothesis of a negative relationship between socioeconomic status and multimorbidity, with contextually different patterns from those observed in the West.

**Methods:**

We conducted our study in the general Hong Kong (HK) population. Data on current health conditions, health behaviours, socio-demographic and socioeconomic characteristics was obtained from HK Government’s Thematic Household Survey. 25,780 individuals aged 15 or above were sampled. Binary logistic and negative binomial regression analyses were conducted to identify risk factors for presence of multimorbidity and number of chronic conditions, respectively. Sub-analysis of possible mediation effect through financial burden borne by private housing residents on multimorbidity was also conducted.

**Results:**

Unadjusted and adjusted models showed that being female, being 25 years or above, having an education level of primary schooling or below, having less than HK$15,000 monthly household income, being jobless or retired, and being past daily smoker were significant risk factors for the presence of multimorbidity and increased number of chronic diseases. Living in private housing was significantly associated with higher chance of multimorbidity and increased number of chronic diseases only after adjustments.

**Conclusions:**

Less advantaged people tend to have higher risks of multimorbidity and utilize healthcare from the public sector with poorer primary healthcare experience. Moreover, middle-class people who are not eligible for government subsidized public housing may be of higher risk of multimorbidity due to psychosocial stress from paying for the severely unaffordable private housing.

## Introduction

Multimorbidity is commonly defined as the co-occurrence of more than one chronic condition within an individual, with different slight modifications across studies for the convenience of analyses [1–3]. Numerous instruments are available for the evaluation of severity in clinical and epidemiological research [4, 5]. A recent systematic review further proposes to include additional connotations to the definition of multimorbidity, including modifiers affecting the burden to the individuals, and the outcomes of multimorbidity [2].

Since the introduction of the concept, most research has focused on healthcare implications and consequences of multimorbidity, rather than the causes and social patterns [6]. Multimorbidity causes higher psychological distress, reduces quality of life, increases use (and hence costs) of primary care, increases hospital admission and increases complication rates in medical interventions [7–13]. In view of these, the social determinants of multimorbidity are worth exploring for several reasons: first, to identify risk factors; second, to understand the mechanism of influence; and third, to map specific levels of intervention and policy entry points [14].

Socioeconomic status is a major determinant of health inequalities [14]. It shapes the intermediary health determinants by producing differences in the experience of exposure and vulnerability to health-compromising conditions, and hence leading to different levels of health status reflective of the individuals’ respective positions within the social hierarchy. Although a number of studies have been conducted to explore the association between several socio-economic indicators such as education, household income, employment status and type of housing in their relationships to multimorbidity, relatively few studies investigated the adjusted relationship between various socioeconomic indicators and multimorbidity altogether [15]. While attempts have been made by researchers to tackle this problem using overall socioeconomic status [16–18], this may cause a substantial loss of information regarding the mechanism of influence on the association since effects of different aspects of the status could not be isolated for analyses [19].

Research of this kind is scarce in Asia [15]. It is especially important to study the association between socioeconomic indicators and multimorbidity in Asia’s developed populations because Asian populations’ pace of socioeconomic development tend to be much more rapid than the traditional, long-term developed Western populations, and thus the patterns of association may differ from those of the Western populations [20]. Moreover, the choice of social groupings to be used for the measurements of health differences is highly contextual [21], and thus, the same socioeconomic indicator may have different health implications in different populations. Hong Kong is one of world’s first populations and the first Chinese population to experience very rapid socioeconomic transition over the past decades. The gross domestic product (GDP) increased from US$ 17,706 million to US$ 197,622 million from 1966 to 2006, while the GDP per capital increased from US$ 4,878 to US$ 28,820 within the same period, ranking at the top in the world [22]. With rapid socioeconomic development, Hong Kong also experienced much faster epidemiologic transition than other Western populations [23]. Therefore, in the present study, we aimed to test how different socioeconomic status indicators were associated with multimorbidity as well as the number of chronic health conditions within the Hong Kong population. We hypothesized that while lower socioeconomic status is generally associated with higher chance of multimorbidity, the patterns of association in Hong Kong may differ from those observed in other long-term developed Western populations.

## Methods

### Data Collection and Study Population

We used data from a large population based survey, the Thematic Household Survey (THS) conducted by the Census and Statistics Department (C&SD) of the Hong Kong SAR Government for the current study. THS provided information on both socioeconomic status indicators and multimorbidity of the population. It is a regular series of territory-wide cross-sectional household surveys covering different social issues since 1999, including health-related topics. The data in the present study was obtained from the October 2011 to January 2012 round of the THS, which collected data on various health-related topics including current health conditions, medical insurance coverage, health care utilization and hospitalization, and also socio-demographic and socioeconomic characteristics [24].

The target population of the THS is the Hong Kong land-based population, excluding institutionalized residents, board vessel residents, foreign domestic helpers and hotel transients. The sampling frame of the survey consisted of all addresses of permanent quarters in built-up areas and records of area segments in non-built-up areas. The latter is deemed necessary because the quarters in these areas may not have clear addresses and cannot readily be identified. The survey covered around 95% of the Hong Kong resident population including both usual and mobile residents.

In this study, 13,411 households were randomly sampled from the discussed sampling frame stratified by district and housing type, within which 10,065 were successfully interviewed face-to-face by trained staff sent to the quarters with structured questionnaires. A total of 29,187 individuals were interviewed. An overall response rate of 75% was achieved. After excluding 3,407 individuals aged under 15 years who were not requested to answer any health-related questions, 25,780 subjects were included for statistical analyses in the study.

### Independent Variables

As recommended by Braveman and colleagues [19], all available and feasible individual indicators of socioeconomic status in relation to the differences in health should be examined so as to reflect the multidimensional nature of socioeconomic status and avoid limitations of standardized measures.

#### Socio-demographic variables

Age was categorized into four groups: ‘15–24,’ ‘25–44,’ ‘45–64,’and ‘65 or above,’ to distinguish between life stages such as adolescence, adulthood, middle age, and late adulthood while gender was included as a dichotomous variable; i.e., male or female.

#### Socioeconomic variables

In this study, we included household income, educational attainment, employment status and type of housing as socioeconomic variables, since they were available variables typically indicative of socioeconomic status from the THS. On the other hand, ethnicity, though usually viewed as an important factor in studies of other populations, was not included as one of the independent variables because the majority (around 94%) of the Hong Kong residential population was ethnic Han Chinese, while other ethnic minorities constitute only less than 2% of the population [25]. It is therefore less of a relevant determinant in Hong Kong than elsewhere.

Information on monthly household income in Hong Kong dollars (HK$1 = US$0.13) was divided into five groups: ‘less than HK$4,000,’ ‘HK$4,000-HK$14,999,’ ‘HK$15,000-HK$24,999,’ ‘HK$25,000- HK$40,000,’ and ‘more than HK$40,000.’ As the median household income of Hong Kong was HK$20,000 in quarter 4 of year 2011, this categorization spans a reasonably wide range of income from the middle point [26].

Education attainment achieved by the individual was classified into ‘post-secondary,’ ‘secondary,’ ‘primary,’ and ‘kindergarten or below.’

Employment status: Employment status was divided into four categories: ‘employed,’ ‘jobless,’ ‘student,’ and ‘retired.’ Individuals who responded that they had a part-time or full-time job in the past seven days were regarded as ‘employed,’ and those who responded otherwise were regarded as ‘jobless,’ excluding ‘students’ who had to attend educational institutions and ‘retired’ persons.

Type of housing consisted of five categories: ‘public,’ ‘subsidized,’ ‘private (tenant),’ ‘private (owner),’ and ‘others.’ According to the latest Census [27] in Hong Kong, 31% resided in public rental housing, 17.8% resided in subsidized sale flats provided by Housing Authority and Housing Society of the Hong Kong Government, 49.3% resided in private housing and 1.9% resided in other types of housing including temporary housing, rooftop structures, mobile dwellings, institutional housing, dormitories, and staff quarters. Ever since the 1950s, public housing has been offered to provide affordable housing for households of low incomes. In other words, residents of public housing do not bear the heavy financial burden from rental expenses or mortgage loan when compared to residents of subsidized or private housing. According to the latest Household Expenditure Survey [28] conducted by the Census and Statistics Department in 2010, an average of 32.8% of the average household expenditure were spent on housing in Hong Kong.

#### Lifestyle factor

Smoking history was included in the analysis and was categorized into two groups: ‘non-smoker,’ and smoker (including ‘occasional smoker,’ ‘past daily smoker,’ ‘past weekly smoker,’ and ‘daily smoker’). Due to data unavailability, we did not include other lifestyle factors such as alcohol consumption as independent factors. Nevertheless, the level of alcohol consumption is traditionally known to be low compared to other countries [29].

#### Outcome Measures

In this study, we used two outcome variables to measure the level of multimorbidity to test for consistency– 1) the presence of two or more chronic health conditions (i.e., the presence of multimorbidity) and 2) the number of chronic health conditions.

Based on self-reported responses, information regarding chronic health conditions as diagnosed by western medical practitioners was collected. Subsequently, the reported health conditions were coded according to a comprehensive list of chronic conditions developed and used by a previous large-scale German study on patterns of multimorbidity where reported diagnoses under the same category were coded as one single condition [30]. Also, we included both concurrent as well as past conditions, because past chronic conditions are highly indicative of later poorer health, and this goes in line with the common view that multimorbidity should be seen as an indicator of health [2, 5, 31]. In our analysis, multimorbidity was defined as suffering from two or more chronic health conditions as in previous similar studies [32]. We used this definition to code a dichotomous variable of the presence of multimorbidity, where only two groups existed, namely: ‘multimorbid’ and ‘non-multimorbid’. On the other hand, the number of chronic health conditions was calculated using the same aforementioned definition and coding of chronic health conditions. Please refer to S1 Appendix for the list of chronic diseases included in this study.

### Statistical Analysis

#### Associations among independent variables

Before examining the association between the socioeconomic variables and multimorbidity, the pairwise bivariate association between independent variables were investigated by constructing a matrix of bivariate relationships. The Cramer’s V statistic was computed for pairs with one or more nominal variables, and the Gamma statistic for pairs of ordinal variables. In addition, generalized variance inflation factor (GVIF), a well-accepted collinearity statistic [33], was calculated to detect the problem of multicollinearity in subsequent multivariable analyses. Also, log-likelihood ratio test were conducted to examine whether the models had any improvement with the inclusion of each additional variables.

#### Presence of multimorbidity

Unadjusted logistic regression analysis was first conducted for each independent variable for an examination of apparent association with multimorbidity, without adjustments for other co-variables. Unadjusted odds ratios and their corresponding 95% confidence intervals were calculated for an illustrative interpretation of results.

We then conducted multivariable logistic regression to examine the association between each independent variable with multimorbidity, with adjustments for other variables. Adjusted odds ratios and their corresponding 95% confidence intervals were calculated to investigate the adjusted association between each independent variable and multimorbidity.

#### Number of chronic health conditions

In addition to the presence of multimorbidity, we also investigated whether the independent variables were associated with the number of chronic conditions. An unadjusted negative binomial regression analysis was conducted to calculate the relative risk and their corresponding 95% confidence intervals for each independent variable.

As for the analysis of the presence of multimorbidity, a further analysis was conducted by constructing an adjusted model with all potential independent variables included altogether, to examine the adjusted association between socioeconomic indicators and number of chronic conditions. Again, relative risk and their corresponding 95% confidence intervals were calculated for an illustrative interpretation of the results.

#### Sub-analysis of possible mediation effect through financial burden

An additional sub-analysis testing possible mediation effect through financial burden on the association between type of housing and outcome (multimorbidity and number of chronic health conditions) was also conducted. While residing in private housing estates is generally viewed as having a better quality of life or having a higher socioeconomic status than residing in subsidized and public housing estates, it is possible that its association with multimorbidity may be mediated by heavier financial burden borne by being private housing residents, which lead to higher burden on paying for the housing and hence more chronic diseases. In order to estimate the financial burden, we used the ratio of total household monthly expenditure over total household monthly income to generate a new proxy variable of financial burden. For both household monthly expenditure and household monthly income, we assigned a continuous score to each categorical level since only categorical data of both variables were collected. A higher score represented a higher income or higher expenditure. By dividing the score on expenditure by that on income, the expenditure-income ratio ranges from 0 (lower financial burden) to 1 (higher financial burden). Subsequently, model-based mediation analysis was conducted to examine the significance of financial burden as a mediator between housing and multimorbidity, and also to provide estimates and confidence intervals of those effects on multimorbidity [34].

Statistical analyses were performed using the computing environment R 3.1.1 [35] and IBM SPSS 21. Multivariate regression analyses were conducted using the R package of ‘mass’ [36] and mediation analysis was applied using another R package, ‘mediation’ [37].

## Results

### Descriptive Statistics (Table 1)

In the present study, 13.5% of the respondents had two or more chronic health conditions (i.e., multimorbidity). More than three percent had three chronic health conditions, and nearly another three percent had four or more. Please refer to Table 1 for more detailed descriptive statistics of the respondents.

**Table 1:** Demographic Characteristics of Respondents.

	N	Percent^a^
**Multimorbidity**		
Non-multimorbid	22553	87.5%
Multimorbid	3227	12.5%
**No. of Chronic Health Conditions**		
0	18200	70.6%
1	4353	16.9%
2	1827	7.1%
3	820	3.2%
≥4	580	2.2%
**Gender**		
Male	12316	47.8%
Female	13464	52.2%
**Age**		
15–24	3967	15.4%
25–44	8356	32.4%
45–64	9253	35.9%
≥65	4204	16.3%
**Education Attainment**		
Kindergarten or below	1460	5.7%
Primary	4573	17.7%
Secondary	15157	58.8%
Post-secondary	4590	17.8%
**Housing Type**		
Private Own	8021	31.1%
Private Rent	2434	9.4%
Subsidized	5806	22.5%
Public	9174	35.6%
Other	345	1.3%
**Household Income**		
<4,000	1410	5.5%
4,000–14,999	5917	23.0%
15,000–24,999	6346	24.6%
25,000–40,000	6372	24.7%
>40,000	5735	22.2%
**Employment Status**		
Employed	14161	54.9%
Jobless	4346	16.9%
Student	2499	9.7%
Retired	4774	18.5%
**Smoking Status**		
Non Smoker#	21707	84.2%
Current/Past Smoker	4073	15.7%

^a^ Percentages of subcategories may not add up to 100% due to rounding of figures.

### Associations among independent variables (Table 2)

The Cramer’s V statistics showed that the pairs of ‘education attainment-employment status’, ‘age-employment status’ and ‘gender-smoking status’ were strongly associated (larger than 0.30). However, age and gender are important covariables in any association model and thus should be included in our regression analyses. Smoking status was the only lifestyle factor in the regression analyses, and thus it would be reasonable to keep it. Also, since the aim of this study was to study the association between different socioeconomic indicators and multimorbidity, and education attainment and employment status could not fully represent each other (Cramer’s V only slightly over 0.30), it was justifiable to keep both variables in our regression analyses. On the other hand, the computed Gamma statistics did not further reveal any strong association between ordinal variables (all below 0.60), and GVIF calculated for each variable were all within an acceptable range, i.e. smaller than 10, implicating that the problem of multicollinearity was unlikely in the subsequent multivariable models. Also, log-likelihood ratio test was statistically significant (p<0.01) at each step of additional variable inclusion.

**Table 2:** Bivariate association between independent variables.

	Employment status	Education attainment	Housing type	Family income	Smoking status	Age
**Employment status**						
**Education attainment**	0.31					
**Housing type**	0.07	0.16				
**Family income**	0.25	0.41*	0.18			
**Smoking status**	0.16	-0.21*	0.10	-0.14*		
**Age**	0.61	-0.58*	0.08	-0.22*	0.22*	
**Gender**	0.27	0.11	0.02	0.03	0.32	0.04

◊ Cramer's V computed for association between a nominal variable and another variable

* Gamma statistic computed for association between ordinal variables.

### Presence of Multimorbidity and Number of Chronic Health Conditions (Table 3)

Table 3 showed the odds ratios and the corresponding 95% confidence intervals of the bivariate and multivariable analysis on the presence of multimorbidity as well as the relative risks and corresponding 95% confidence intervals of the bivariate and multivariable analysis on the number of chronic health conditions.

**Table 3:** Results of regression analyses^a^.

	Presence of Multimorbidity (Binary Logistic)						Number of Chronic Conditions (Negative Binomial)					
	Unadjusted (bivariate)			Adjusted for all covariables			Unadjusted (bivariate)			Adjusted for all covariables		
	OR	95% C.I.	Sig.	OR	95% C.I.	Sig.	RR	95% C.I.	Sig.	RR	95% C.I.	Sig.
**Gender**												
Female	1.195	1.109–1.287	0.000	1.139	1.084–1.197	0.008	1.123	1.095–1.152	0.000	1.048	1.022–1.074	0.061
**Age**												
15–24	1			1			1			1		
25–44	2.145	1.601–2.874	0.000	1.695	1.381–2.080	0.010	1.331	1.193–1.484	0.000	1.300	1.201–1.407	0.001
45–64	9.971	7.605–13.073	0.000	6.297	5.164–7.680	0.000	4.304	3.896–4.753	0.000	3.579	3.315–3.864	0.000
≥ 65	50.589	38.588–66.323	0.000	18.359	14.935–22.568	0.000	12.043	10.885–13.324	0.000	6.610	6.083–7.182	0.000
**Education**												
Post-secondary	1			1			1			1		
Secondary	1.231	1.079–1.406	0.002	.953	0.884–1.027	0.522	1.123	1.050–1.202	0.001	0.927	0.894–0.961	0.037
Primary	4.808	4.196–5.509	0.000	1.287	1.185–1.398	0.002	3.049	2.836–3.279	0.000	1.140	1.094–1.189	0.002
Kindergarten or below	11.163	9.539–13.064	0.000	1.689	1.533–1.859	0.000	5.019	4.593–5.485	0.000	1.300	1.236–1.366	0.000
**Housing**												
Public	1			1			1			1		
Subsidized	0.832	0.754–0.919	0.000	1.111	1.048–1.178	0.070	0.834	0.788–0.883	0.000	0.993	0.963–1.023	0.816
Private (Tenant)	0.681	0.588–0.788	0.000	1.187	1.092–1.292	0.041	0.760	0.702–0.824	0.000	1.116	1.070–1.164	0.009
Private (Owner)	0.887	0.812–0.970	0.008	1.173	1.111–1.239	0.003	0.923	0.878–0.971	0.002	1.102	1.071–1.133	0.001
Others	0.614	0.423–0.893	0.011	.706	0.572–0.871	0.097	0.680	0.555–0.834	0.000	0.775	0.697–0.861	0.016
**Household Income**												
>HKD40,000	1			1			1			1		
HKD25,000–40,000	1.232	1.083–1.403	0.002	1.072	0.997–1.152	0.339	1.066	0.995–1.142	0.068	0.954	0.920–0.989	0.187
HKD15,000–24,999	1.313	1.155–1.492	0.000	.986	0.916–1.060	0.843	1.208	1.129–1.292	0.000	0.977	0.943–1.013	0.527
HKD4,000–14,999	2.837	2.523–3.190	0.000	1.433	1.336–1.537	0.000	2.065	1.937–2.202	0.000	1.184	1.142–1.227	0.000
<4,000	5.870	5.063–6.807	0.000	1.522	1.393–1.662	0.000	3.360	3.080–3.665	0.000	1.214	1.159–1.272	0.000
**Employment Status**												
Employed	1			1			1			1		
Jobless	2.690	2.409–3.004	0.000	1.639	1.537–1.748	0.000	2.078	1.956–2.208	0.000	1.453	1.406–1.502	0.000
Student	0.210	0.147–0.299	0.000	0.817	0.631–1.059	0.435	0.462	0.409–0.522	0.000	1.113	1.014–1.222	0.248
Retired	9.355	8.535–10.253	0.000	1.764	1.646–1.891	0.000	4.859	4.616–5.116	0.000	1.594	1.536–1.654	0.000
**Smoking Status**												
Non-smoker	1			1			1			1		
Current/Past Smoker	1.439	1.372–1.509	0.000	1.367	1.288–1.450	0.000	1.301	1.260–1.345	0.000	1.208	1.172–1.246	0.000

^a^ Forward model construction followed this sequence: demographics -> income -> education -> housing -> employment status, with log-likelihood ratio test result being statistically significant at every step for both models (p<0.01).

In the bivariate analysis for the presence of multimorbidity, being female, being 25 years old or above, having less than post-secondary education level, having less than HK$40,000 monthly household income, being jobless or retired, and smoking were statistically significant factors associated with the presence of multimorbidity. There were also apparent dose-response relationships between multimorbidity with age, education level, and household income. Residing in non-public housing and being a student were negatively associated with the presence of multimorbidity. The results for the number of chronic health conditions were generally consistent with those for the presence of multimorbidity—being female, being 25 years or above, having less than post-secondary education level, having less than HK$25,000 monthly household income, being jobless or retired, and smoking were all statistically significant risk factors for increased number of chronic conditions. Similar to the presence of multimorbidity, dose-response relationships with increased number of chronic conditions were also observed for age, education level and household income. Also consistent with the results for the presence of multimorbidity, residing in non-public housing and being a student were negatively associated with the increased number of chronic conditions.

In the analysis for the presence of multimorbidity after adjustments for other co-variables, the direction of association remained the same for most factors—i.e., being female, being 25 years or above, having an education level of primary schooling or below, having less than HK$15,000 monthly household income, being jobless or retired, and smoking remained to be significant risk factors for multimorbidity. However, the direction of association with multimorbidity became reversed for housing types. In particular, residing in private owned or rental housing became significant risk factors for multimorbidity after adjusting for other variables. After adjustments, only age still showed significant dose-response relationship with multimorbidity. Similarly in the analysis for the number of chornic conditions after adjustments for other covariables, the direction of association remained similar for most factors—i.e., being 25 years or above, having an education level of secondary schooling or below, having less than HK$15,000 monthly household income, being jobless or retired, and smoking remained to be significant risk factors for increased number of chronic diseases. Also consistent with the results for the presence of multimorbidity, the direction of association with increased number of chronic diseases was reversed for housing types after adjustments. Residing in private owned or rental housing again became significant risk factors for increased number of chronic diseases after adjustments. Also, age remained to show significant dose-response relationship with increased number of chronic diseases after adjustments.

Both logistic and negative binomial regression analyses were also conducted separately for male and female, as well as for the four different age groups. Similar results were identified.

### Possible mediation effect of financial burden (Table 4)

As shown in Table 4, statistically significant, albeit small, mediation effect of financial burden (proxied by the simulated expenditure/income ratio) acting between housing type and multimorbidity was evidently supported by the data. It was shown that 1.36% (1.46%) of the association of non-private housing (private housing) with the presence of multimorbidity was significantly mediated through increasing financial burden. Similarly, for number of chronic diseases, 1.15% (1.31%) were the proportion mediated through increasing financial burden. On average, around 1.41% of the association of housing type with the presence of multimorbidity was significantly mediated through increasing financial burden, while 1.24% of that association with the number of chronic health conditions was mediated similarly.

**Table 4:** Results of meditation analyses.

	Estimate	95% CI	p-value
**Presence of Multimorbidity**			
ACME (non-private housing)	0.0002	0.0000–0.0004	0.010
ACME (private housing)	0.0002	0.0000–0.0004	0.010
ADE (non-private housing)	0.0132	0.0054–0.0207	0.000
ADE (private housing)	0.0132	0.0054–0.0207	0.000
Total Effect	0.0134	0.0056–0.0208	0.000
Proportion Mediated (non-private housing)	1.36%	0.24%-4.00%	0.010
Proportion Mediated (private housing)	1.46%	0.27%-4.20%	0.010
ACME (average)	0.0002	0.0000–0.0004	0.010
ADE (average)	0.0132	0.0054–0.0207	0.000
Proportion Mediated (average)	1.41%	0.26%-4.08%	0.010
**Number of Chronic Health Conditions**			
ACME (non-private housing)	0.0008	0.0003–0.0013	0.000
ACME (private housing)	0.0009	0.0003–0.0015	0.000
ADE (non-private housing)	0.0642	0.0393–0.0894	0.000
ADE (private housing)	0.0643	0.0393–0.0895	0.000
Total Effect	0.0650	0.0399–0.0902	0.000
Proportion. Mediated (non-private housing)	1.16%	0.37%-2.57%	0.000
Proportion. Mediated (private housing)	1.31%	0.43%-2.81%	0.000
ACME (average)	0.0008	0.0003–0.0014	0.000
ADE (average)	0.0642	0.0393–0.0895	0.000
Proportion Mediated (average)	1.24%	0.40%-2.69%	0.000

Sample Size Used: 25780

Simulations: 1000

ACME = Average Causal Mediation Effects

ADE = Average Direct Effects.

## Discussion

### Summary

In general, our hypothesis that socioeconomic status is inversely related to multimorbidity and number of chronic health conditions is empirically supported by the results. Among the four indicators of socioeconomic status, household income, educational attainment and employment status were shown to have negative associations with the outcome variables (i.e. the presence of multimorbidity and the number of chronic diseases). On the contrary, we also consistently found that living in private housing was significantly associated with higher chance of multimorbidity and increased number of chronic diseases, with slight mediation effect through financial burden.

### Limitations

Nevertheless, notwithstanding the population representativeness of the THS survey, there are limitations. First, all data, including the health conditions, were self-reported, which might lead to bias and inaccuracy in recalling and reporting. The prevalence of multimorbidity is low in the present study compared to other studies. In this case, it is likely that the less advantaged might have generally use western medical practitioners in the outpatient clinics less often, since most people visit private western medical practitioners for outpatient consultations in Hong Kong. Thus, the levels of multimorbidity might have been underestimated. Also, as all data were self-reported, there could also be an association between one’s socioeconomic status and the number of diseases a person can remember or recall. We should be aware of this limitation when interpreting our results. Second, the definition of multimorbidity, as previously mentioned, has been under ongoing debates and modifications; hence, using different definitions might give different results in the analyses. The version we adopted in the present study has also been criticized as being overly simplistic [3, 5]. However, many previous studies have adopted this definition, and the use of it could enhance the comparability and interpretability of the results [5]. Third, the cross-sectional nature of the dataset renders any causal inferences implausible. It is nevertheless an important first step in the exploration of social determinants of multimorbidity in Hong Kong which could shed light on future studies of similar kinds and help identify entry points for public health policies for the government. Social strata that are in need of additional resources could also be easily recognized according to the results of this study; thus, a general social pattern of multimorbidity is identified. Fourth, due to data availability, we did not include other determining factors of multimorbidity in our models, such as other lifestyle factors other than smoking, family history of chronic conditions, the use of medications and clinical history. Nevertheless, there is no reason why these factors would be unevenly distributed across the socioeconomic spectrum. In addition, we did not use all available socioeconomic indicators for the analyses. Although information on respondents’ occupational position is available in the THS, the classification is too ambiguous for analysis. For example, the options ‘professional’ and ‘associate professional’ could be difficult for respondents to distinguish. Besides, the results of the rest of the models seem indifferent to the presence of occupational position in numerous trials. We therefore decided not to include occupational position. Last, there may also be bias in the sample towards the younger age groups who tend to reside in relatively newly developed districts of the city. Apart from the age effects, the district they lived in might have played an important role in determining their health via the variation in quality of the environment. Nevertheless, due to the absence of available district data, we did not include that for adjustment. Future studies should place more emphasis on that as a determinant of multimorbidity or even overall health status.

### Relationship with Existing Literature

The present study obtained similar results with the existing literature [16, 17, 38–45]. For instance, educational attainment and income were consistently found to be negatively related to the presence of multimorbidity in various populations, mostly European [39–43, 46]. Nonetheless, this study is the first of its kind in a Chinese population. On the other hand, residing in private housing, albeit a conventionally indicator of a higher SES [47], appeared to be positively associated with multimorbidity and increased number of chronic diseases after adjustments for other variables. This differed from the results of previous studies [17, 32, 43, 48] where residents in deprived areas were found to be associated with multimorbidity and more chronic health conditions.

### Explanation of Findings

Household income, education attainment and employment status still remained to be significant, albeit weaker, risk factors of multimorbidity after adjustments for other variables, implying that they were individually associated with multimorbidity and number of chronic health conditions.

On the other hand, the different patterns of association between housing type and multimorbidity observed in Hong Kong versus in previous studies can be explained by several possible reasons. First, previous studies [32, 48] used neighborhood-level deprivation index as the independent variable, which was a more composite measure that consists of several socioeconomic factors, and did not distinguish the association between residence area and multimorbidity, as independent to that of income, age and education level with multimorbidity. Second, residing in public housing in Hong Kong is a completely different experience from residing in deprived areas in the United Kingdom. Public housing do not necessarily situate in the more socially deprived areas in Hong Kong. In fact, in Hong Kong, public housing estates are rather evenly distributed throughout the land, and some public housing estates situate themselves in the richest areas of Hong Kong. On other hand, as mentioned earlier, residents of public housing in general do not bear the heavy financial burden from rental expenses or mortgage loan when compared to residents of subsidized or private housing. Hong Kong ranks number one in the world for most unaffordable housing—it would take 14.9 years for a household earning median household income to pay for the median housing price, which is well above the second-place Vancouver (10.3 years) and other developed cities including London (7.7 years) and Singapore (5.1 years) [49]. This may have significant psychosocial impact on health, especially for the private housing residents, leading to a higher chance of multimorbidity [50, 51]. Also, the follow-up analysis we conducted on the mediation effect of the financial burden between private housing resident status and multimorbidity served as additional empirical ground on which we based our speculation.

Nevertheless, this warrants further studies to verify, partly because our analysis was limited in the sense that the proxy of financial burden was calculated using total household expenditure instead of housing expenditure, and was therefore not an exact measure of the housing stress. Moreover, the proxy of financial burden only represented the current financial situation instead of the accumulated burden throughout the years. Both limitations may contribute to why the proportion of mediated effect was low. It is therefore recommended that future studies to include variables on financial situation and patterns of expenditures and savings of individuals so that the adjusted association between the type of housing and multimorbidity could be further investigated.

### Public Health Implications

Our findings showed that people in lower socioeconomic position (as proxied by monthly household income, education attainment and employment status) tended to have higher risks for multimorbidity and increased number of chronic diseases and therefore were in need of medical care more than their counterparts with higher socioeconomic position. Hong Kong has a mixed healthcare system with both public and private sectors, where the public healthcare services are largely paid by the general taxation with additional low fees at the point-of-care, while the private healthcare service are largely paid out-of-pocket in addition to private medical insurance and employer-provided medical benefits if available [52]. This design of the healthcare system is in line with Hong Kong’s general healthcare principle that “no one should be denied adequate medical treatment through lack of means”[53] so that the less advantaged individuals can access affordable healthcare from the public sector. Nevertheless, a previous study in Hong Kong [54] showed that patients of higher social status has had better primary care experiences which included better access and continuity of care when compared to those with lower social status. As previous research has suggested the important role of primary care in the management of multimorbidity [3], findings from the present study and the study by Wong et al [54] suggest that people with lower socio-economic status, although having a higher burden of multimorbidity, may receive less access and continuity of care as compared to those of higher status but with less burden of diseases.

## Conclusion

In this study, we found that less advantaged people tend to have higher risks of multimorbidity and utilize healthcare from the public sector with poorer primary healthcare experience in Hong Kong. This warrants future healthcare practice to pay more attention to the social determinants of health. Moreover, middle-class people who are not eligible for government subsidized public housing may be of higher risk of multimorbidity possibly due to psychosocial stress from paying for the severely unaffordable private housing. Since little is known about the specific mechanism by which socioeconomic determinants affects multimorbidity, more studies on the mediation by intermediary (e.g. psychosocial, lifestyle and materialistic determinants) on the association between socioeconomic status and multimorbidity are needed to gain a better understanding of social production of health. Longitudinal studies are also needed to establish temporality for the causal relationship between socioeconomic factors and multimorbidity. Studies should also be conducted in other parts of China to test for external validity.

## Supporting Information

S1 Appendix List of the 46 chronic conditions used in this study and their corresponding ICD codes. ICD = International Classification of Diseases (10th edition).
(DOCX)

## References

[1] van den Akker M, Buntinx F, Knottnerus JA. Comorbidity or multimorbidity. European Journal of General Practice. 1996;2(2):65–70. 10.3109/13814789609162146

[2] Le Reste JY, Nabbe P, Manceau B, Lygidakis C, Doerr C, Lingner H, et al The European General Practice Research Network Presents a Comprehensive Definition of Multimorbidity in Family Medicine and Long Term Care, Following a Systematic Review of Relevant Literature. Journal of the American Medical Directors Association. 2013;14(5):319–25. 10.1016/j.jamda.2013.01.001. 10.1016/j.jamda.2013.01.001 23411065

[3] Mercer SW, Smith SM, Wyke S, O'Dowd T, Watt GC. Multimorbidity in primary care: developing the research agenda. Family Practice. 2009;26(2):79–80. 10.1093/fampra/cmp020 19287000

[4] de Groot V, Beckerman H, Lankhorst GJ, Bouter LM. How to measure comorbidity: a critical review of available methods. Journal of Clinical Epidemiology. 2003;56(3):221–9. 10.1016/S0895-4356(02)00585-1. 12725876

[5] Diederichs C, Berger K, Bartels DB. The Measurement of Multiple Chronic Diseases—A Systematic Review on Existing Multimorbidity Indices. The Journals of Gerontology Series A: Biological Sciences and Medical Sciences. 2011;66A(3):301–11. 10.1093/gerona/glq208 21112963

[6] Gijsen R, Hoeymans N, Schellevis FG, Ruwaard D, Satariano WA, van den Bos GAM. Causes and consequences of comorbidity: A review. Journal of Clinical Epidemiology. 2001;54(7):661–74. 10.1016/S0895-4356(00)00363-2. 11438406

[7] Fortin M, Lapointe L, Hudon C, Vanasse A, Ntetu AL, Maltais D. Multimorbidity and quality of life in primary care: a systematic review. Health and Quality of life Outcomes. 2004;2(1):51. 15380021 10.1186/1477-7525-2-51 PMC526383

[8] Fortin M, Bravo G, Hudon C, Lapointe L, Dubois M-F, Almirall J. Psychological Distress and Multimorbidity in Primary Care. The Annals of Family Medicine. 2006;4(5):417–22. 10.1370/afm.528 17003141 PMC1578652

[9] Fortin M, Dubois MF, Hudon C, Soubhi H, Almirall J. Multimorbidity and quality of life: a closer look. Health and quality of life outcomes. 2007;5:52 Epub 2007/08/09. 10.1186/1477-7525-5-52 ; PubMed Central PMCID: PMCPmc2042974. 17683600 PMC2042974

[10] Campbell-Scherer D. Multimorbidity: a challenge for evidence-based medicine. Evidence Based Medicine. 2010;15(6):165–6. 10.1136/ebm1154 21106673

[11] Glynn LG, Valderas JM, Healy P, Burke E, Newell J, Gillespie P, et al The prevalence of multimorbidity in primary care and its effect on health care utilization and cost. Family Practice. 2011;28(5):516–23. 10.1093/fampra/cmr013 21436204

[12] van Oostrom S, Picavet HS, de Bruin S, Stirbu I, Korevaar J, Schellevis F, et al Multimorbidity of chronic diseases and health care utilization in general practice. BMC Family Practice. 2014;15(1):61 10.1186/1471-2296-15-61 24708798 PMC4021063

[13] Payne RA, Abel GA, Guthrie B, Mercer SW. The effect of physical multimorbidity, mental health conditions and socioeconomic deprivation on unplanned admissions to hospital: a retrospective cohort study. Canadian Medical Association Journal. 2013;185(5):E221–E8. 10.1503/cmaj.121349 23422444 PMC3602270

[14] Solar O, Irwin A. A conceptual framework for action on the social determinants of health. Geneva: World Health Organization; 2010.

[15] Marengoni A, Angleman S, Melis R, Mangialasche F, Karp A, Garmen A, et al Aging with multimorbidity: A systematic review of the literature. Ageing Research Reviews. 2011;10(4):430–9. 10.1016/j.arr.2011.03.003. 10.1016/j.arr.2011.03.003 21402176

[16] Violan C, Foguet-Boreu Q, Roso-Llorach A, Rodriguez-Blanco T, Pons-Vigues M, Pujol-Ribera E, et al Burden of multimorbidity, socioeconomic status and use of health services across stages of life in urban areas: a cross-sectional study. BMC Public Health. 2014;14(1):530 10.1186/1471-2458-14-530 24885174 PMC4060853

[17] Salisbury C, Johnson L, Purdy S, Valderas JM, Montgomery AA. Epidemiology and impact of multimorbidity in primary care: a retrospective cohort study. British Journal of General Practice. 2011;61(582):e12–e21. 10.3399/bjgp11X548929 21401985 PMC3020068

[18] Orueta J, Nuño-Solinís R, García-Alvarez A, Alonso-Morán E. Prevalence of multimorbidity according to the deprivation level among the elderly in the Basque Country. BMC Public Health. 2013;13(1):1–11. 10.1186/1471-2458-13-918 24088559 PMC3852493

[19] Braveman PA, Cubbin C, Egerter S, et al Socioeconomic status in health research: One size does not fit all. JAMA. 2005;294(22):2879–88. 10.1001/jama.294.22.2879 16352796

[20] Schooling CM, Leung GM. A socio-biological explanation for social disparities in non-communicable chronic diseases: the product of history? Journal of epidemiology and community health. 2010;64(11):941–9. Epub 2010/06/03. 10.1136/jech.2008.086553 . 20515893

[21] Kawachi I, Subramanian SV, Almeida-Filho N. A glossary for health inequalities. Journal of epidemiology and community health. 2002;56(9):647–52. Epub 2002/08/15. ; PubMed Central PMCID: PMCPmc1732240. 12177079 10.1136/jech.56.9.647 PMC1732240

[22] Hong Kong Census and Statistics Department. Hong Kong Statistics. Hong Kong: Government of the Hong Kong Administrative Region; 2010.

[23] Chung RY, Leung GM, Cowling BJ, Schooling CM. Patterns of and hypotheses for infection-related cancers in a Chinese population with rapid economic development. Epidemiology and infection. 2012;140(10):1904–19. Epub 2011/12/07. 10.1017/s0950268811002469 . 22142566

[24] Hong Kong Census and Statistics Department. Thematic Household Survey Report—Report No. 50. Hong Kong: Hong Kong Special Administration Region Government, 2013.

[25] Department HA. The Demographics: Ethnic Groups Hong Kong: Hong Kong Special Adminitration Region Government; 2013 [updated 30 July 2013; cited 2014 Oct 26]. Available from: http://www.had.gov.hk/rru/english/info/info_dem.html.

[26] Hong Kong Census and Statistics Department. Quarterly Report on General Household Survey (Fourth Quarter 2011). Hong Kong: Government of the Hong Kong Administrative Region; 2012.

[27] Hong Kong Census and Statistics Department. Table 1960: Population by Type of Housing, 1996, 2001 and 2006. Hong Kong: Hong Kong Census and Statistics Department, Hong Kong Special Administrative Region Government; 2006.

[28] Hong Kong Census and Statistics Department. Table 54C: 2009/10 Household Expenditure Survey—Average Monthly Household Expenditure by Commodity/Service Section/Group. Hong Kong: Hong Kong Census and Statistics Department, Hong Kong Special Administrative Region Government; 2011.

[29] Chung RY, Kim JH, Yip BH, Wong SYS, Wong MCS, Chung VCH, et al Alcohol Tax Policy and Related Mortality. An Age-Period-Cohort Analysis of a Rapidly Developed Chinese Population, 1981–2010. PLoS ONE. 2014;9(8):e99906 10.1371/journal.pone.0099906 25153324 PMC4143164

[30] van den Bussche H, Koller D, Kolonko T, Hansen H, Wegscheider K, Glaeske G, et al Which chronic diseases and disease combinations are specific to multimorbidity in the elderly? Results of a claims data based cross-sectional study in Germany. BMC Public Health. 2011;11(1):101 10.1186/1471-2458-11-101 21320345 PMC3050745

[31] Habib RR, Hojeij S, Elzein K, Chaaban J, Seyfert K. Associations between life conditions and multi-morbidity in marginalized populations: the case of Palestinian refugees. European journal of public health. 2014;24(5):727–33. Epub 2014/07/06. 10.1093/eurpub/cku089 ; PubMed Central PMCID: PMCPmc4168045. 24994504 PMC4168045

[32] Mercer SW, Watt GCM. The Inverse Care Law: Clinical Primary Care Encounters in Deprived and Affluent Areas of Scotland. The Annals of Family Medicine. 2007;5(6):503–10. 10.1370/afm.778 18025487 PMC2094031

[33] Fox J, Monette G. Generalized Collinearity Diagnostics. Journal of the American Statistical Association. 1992;87(417):178–83. 10.2307/2290467 12155413

[34] IMAI K, KEELE L, TINGLEY D, YAMAMOTO T. Unpacking the Black Box of Causality: Learning about Causal Mechanisms from Experimental and Observational Studies. American Political Science Review. 2011;105(04):765–89. 10.1017/S0003055411000414

[35] R Core Team. R: A Language and Environment for Statistical Computing. 2014.

[36] Ripley B, Venables B, Bates D, Hornik K, Gebhardt A, Firth D. Support Functions and Datasets for Venables and Ripley's MASS: The Comprehensive R Archive Network; 2014 [cited 2014 September 3]. Available from: http://cran.r-project.org/web/packages/MASS/MASS.pdf.

[37] Tingley D, Yamamoto T, Hirose K, Keele L, Imai K. Causal Mediation Analysis: The Comprehensive R Archive Network; 2014 [cited 2014 September 3]. Available from: http://cran.r-project.org/web/packages/mediation/mediation.pdf.

[38] Nagel G, Peter R, Braig S, Hermann S, Rohrmann S, Linseisen J. The impact of education on risk factors and the occurrence of multimorbidity in the EPIC-Heidelberg cohort. BMC Public Health. 2008;8(1):384 10.1186/1471-2458-8-384 19014444 PMC2614432

[39] Andrade LH, Benseñor IM, Viana MC, Andreoni S, Wang Y-P. Clustering of psychiatric and somatic illnesses in the general population: multimorbidity and socioeconomic correlates. Brazilian Journal of Medical and Biological Research. 2010;43:483–91. 20379689 10.1590/s0100-879x2010007500024

[40] Tucker-Seeley R, Li Y, Sorensen G, Subramanian SV. Lifecourse socioeconomic circumstances and multimorbidity among older adults. BMC Public Health. 2011;11(1):1–9. 10.1186/1471-2458-11-313 21569558 PMC3118239

[41] Agborsangaya C, Lau D, Lahtinen M, Cooke T, Johnson J. Multimorbidity prevalence and patterns across socioeconomic determinants: a cross-sectional survey. BMC Public Health. 2012;12(1):1–8. 10.1186/1471-2458-12-201 22429338 PMC3353224

[42] Schäfer I, Hansen H, Schön G, Höfels S, Altiner A, Dahlhaus A, et al The influence of age, gender and socio-economic status on multimorbidity patterns in primary care. first results from the multicare cohort study. BMC Health Serv Res. 2012;12(1):1–15. 10.1186/1472-6963-12-89 22471952 PMC3348059

[43] Alaba O, Chola L. The social determinants of multimorbidity in South Africa. Int J Equity Health. 2013;12(1):1–10. 10.1186/1475-9276-12-63 23962055 PMC3846856

[44] Ataguba JE-O. Inequalities in multimorbidity in South Africa. International Journal for Equity in Health. 2013;12(1):64 10.1186/1475-9276-12-64 23962076 PMC3765402

[45] Chau K, Baumann M, Chau N. Socioeconomic inequities patterns of multi-morbidity in early adolescence. International Journal for Equity in Health. 2013;12(1):1–12. 10.1186/1475-9276-12-65 23962097 PMC3765191

[46] van den Akker M, Buntinx F, Metsemakers JFM, Roos S, Knottnerus JA. Multimorbidity in General Practice: Prevalence, Incidence, and Determinants of Co-Occurring Chronic and Recurrent Diseases. Journal of clinical epidemiology. 1998;51(5):367–75. 9619963 10.1016/s0895-4356(97)00306-5

[47] Lee J, Ngai-ming Y. Public Housing and Family Life in East Asia: Housing History and Social Change in Hong Kong, 1953–1990. Journal of Family History. 2006;31(1):66–82. 10.1177/0363199005283008

[48] Barnett K, Mercer SW, Norbury M, Watt G, Wyke S, Guthrie B. Epidemiology of multimorbidity and implications for health care, research, and medical education: a cross-sectional study. The Lancet. 2012;380(9836):37–43. 10.1016/S0140-6736(12)60240-2 22579043

[49] Bertaud A. 10th Annual Demographia International Housing Affordability Survey: 2014. Belleville, Illinois: 2014.

[50] Matthews KA, Gallo LC, Taylor SE. Are psychosocial factors mediators of socioeconomic status and health connections? Annals of the New York Academy of Sciences. 2010;1186(1):146–73. 10.1111/j.1749-6632.2009.05332.x 20201872

[51] Matthews KA, Gallo LC. Psychological perspectives on pathways linking socioeconomic status and physical health. Annual review of psychology. 2011;62:501–30. Epub 2010/07/20. 10.1146/annurev.psych.031809.130711 ; PubMed Central PMCID: PMCPmc3121154. 20636127 PMC3121154

[52] Leung GM, Wong IO, Chan WS, Choi S, Lo SV. The ecology of health care in Hong Kong. Social science & medicine (1982). 2005;61(3):577–90. Epub 2005/05/19. 10.1016/j.socscimed.2004.12.029 . 15899317

[53] Food and Health Bureau. Healthcare reform consultation document—your health your life. Hong Kong: Government of the Hong Kong Administrative Region; 2008.

[54] Wong S, Kung K, Griffiths S, Carthy T, Wong M, Lo S, et al Comparison of primary care experiences among adults in general outpatient clinics and private general practice clinics in Hong Kong. BMC Public Health. 2010;10(1):1–11. 10.1186/1471-2458-10-397 20602806 PMC2908092

