# Supplementary material for: Socioeconomic Determinants of Multimorbidity: A Population-Based Household Survey of Hong Kong Chinese
Source: PLoS One. 2015 Oct 9;10(10):e0140040. doi: 10.1371/journal.pone.0140040 (PMC4599799; doi:10.1371/journal.pone.0140040)
Supplement: S1 Appendix — ICD = International Classification of Diseases (10th edition). (DOCX) [file pone.0140040.s001.docx]

**Appendix. List of the 46 chronic conditions used in this study and their corresponding ICD codes**

| **No** | **Chronic condition** | **ICD-10 codes** |
| --- | --- | --- |
| 1 | Hypertension | I10-I15 |
| 2 | Lipid metabolism disorders | E78 |
| 3 | Chronic low back pain | M40-M45, M47, M48.0-M48.2, M48.5-M48.9, M50-M54 |
| 4 | Severe vision reduction | H17-H18, H25-H28, H31, H33, H34.1-H34.2, H34.8-H34.9, H35-H36,  H40, H43, H47, H54 |
| 5 | Osteoarthrosis | M15-M19 |
| 6 | Diabetes mellitus | E10-E14 |
| 7 | Chronic ischemic heart disease | I20, I21, I25 |
| 8 | Thyroid dysfunction | E01-E05, E06.1-E06.3, E06.5, E06.9, E07 |
| 9 | Cardiac arrhythmias | I44-I45, I46.0, I46.9, I47-I48, I49.1-I49.9 |
| 10 | Obesity | E66 |
| 11 | Purine/pyrimidine metabolism  disorders/Gout | E79, M10 |
| 12 | Prostatic hyperplasia | N40 |
| 13 | Lower limb varicosis | I83, I87.2 |
| 14 | Liver disease | K70, K71.3-K71.5, K71.7, K72.1, K72.7, K72.9, K73-K74, K76 |
| 15 | Depression | F32-F33 |
| 16 | Asthma/COPD | J40-J45, J47 |
| 17 | Noninflammatory gynecological  Problems | N81, N84-N90, N93, N95 |
| 18 | Atherosclerosis/PAOD | I65-I66, I67.2, I70, I73.9 |
| 19 | Osteoporosis | M80-M82 |
| 20 | Renal insufficiency | N18-N19 |
| 21 | Cerebral ischemia/Chronic  Stroke | I60-I64, I69, G45 |
| 22 | Cardiac insufficiency | I50 |
| 23 | Severe hearing loss | H90, H91.0, H91.1, H91.3, H91.8, H91.9 |
| 24 | Chronic cholecystitis/Gallstones | K80, K81.1 |
| 25 | Somatoform disorders | F45 |
| 26 | Hemorrhoids | I84 |
| 27 | Intestinal diverticulosis | K57 |
| 28 | Rheumatoid arthritis/Chronic  Polyarthritis | M05-M06, M79.0 |
| 29 | Cardiac valve disorders | I34-I37 |
| 30 | Neuropathies | G50-G64 |
| 31 | Dizziness | H81-H82, R42 |
| 32 | Dementia | F00-F03, F05.1, G30, G31, R54 |
| 33 | Urinary incontinence | N39.3-N39.4, R32 |
| 34 | Urinary tract calculi | N20 |
| 35 | Anemia | D50-D53, D55-D58, D59.0-D59.2, D59.4-D59.9, D60.0, D60.8, D60.9,  D61, D63-D64 |
| 36 | Anxiety | F40-F41 |
| 37 | Psoriasis | L40 |
| 38 | Migraine/chronic headache | G43, G44 |
| 39 | Parkinson’s disease | G20-G22 |
| 40 | Cancers | C00-C14, C15-C26, C30-C39, C40-C41, C43-C44, C45-C49, C50,  C51-C58, C60-C63, C64-C68, C69-C72, C73-C75, C81-C96, C76-  C80, C97, D00-D09, D37-D48 |
| 41 | Allergies | H01.1, J30, L23, L27.2, L56.4, K52.2, K90.0, T78.1, T78.4, T88.7 |
| 42 | Chronic gastritis/GERD | K21, K25.4-K25.9, K26.4-K26.9, K27.4-K27.9, K28.4-K28.9, K29.2-  K29.9 |
| 43 | Sexual dysfunction | F52, N48.4 |
| 44 | Insomnia | G47, F51 |
| 45 | Tobacco abuse | F17 |
| 46 | Hypotension | I95 |

ICD = International Classification of Diseases (10th edition)
